# 

**Published:** 1912-01

**Authors:** 


### BUFFALO MEDICAL JOURNAL

**A Monthly Review of Medicine and Surgery**

*EDITOR*

A. L. BENEDICT.

All communications, whether of a literary or business nature, books for reviewand exchanges, should be addressed to the editor, 354 FRANKLIN STREET, BUFFALO, N. Y. Please make personal or telephonic calls before 1 p. m

THE BUFFALO MEDICAL JOURNAL will publish regularly. full transactions of any professional organization in western New York at cost. Personal notices, brief reports of society proceedings, and the like, are always welcome and will be published without charge.

Subscriptions may commence at any time. $2.00 per year in advance,

VOL. LXVII. JANUARY, 1912 No. 6

*It is better to be a climber, with scarred shins and tibial exostoses, than to have callosities over the ischial tuberosities from being boosted.*

### Announcements

Any one who wishes copies of the BUFFALO MEDICAL JOURNAL containing his own contribution, or for any other reason, may have them for the asking. We have about ten copies of nearly every number since 1885 and wish to condense our files. Files, as nearly complete as possible, will also be furnished for libraries.

Will you do your part toward making advertisers understand that circulars are a nuisance and a waste of money and effort? Will you help them to realize that legitimate advertising in periodicals is cheap and effective? We are not asking this from any narrowly selfish policy. We do not refer in particular to this JOURNAL, nor merely to medical journals in general but to the whole range of periodicals from the penny newspaper to the literary magazine so aristocratic that there is no club rate on it. Every advertising circular read, adds to the cost and detracts from the possible excellence of every bona fide newspaper, literary and technical magazine.

WILL our readers, especially those who are officers or high privates in medical societies, consider carefully and solely in the interest of the societies, our standing offer with regard to publication of proceedings?

Aside from the regular associate staff, representing the larger cities of western New York, we should like to have a correspondent in each city and village which has a medical population of a dozen or more. The duties of the correspondent would be, on a smaller scale, precisely the same as for associate editors —to see that the activities and interests of their locality are properly represented in the JOURNAL and, incidentally to look after the interests of the JOURNAL. The position is highly honorable, not at all onerous and, it must be confessed, not very lucrative, though we shall try to show our appreciation in various minor perquisits. Don't hesitate to volunteer.

For that matter, we should like to have every subscriber send us word of his own doings from time to time, note deaths and other occurrences in his neighborhood and tell his confreres what the JOURNAL is doing.

---

READ—and heed—the advertisements: By so doing you will get a better and larger JOURNAL. If the reason is not apparent, we will explain.

---

WE have a number of general and special journals, in various languages, which we would like to turn over to anyone who would review them systematically and skim off the cream for our readers. In particular, we have a most interesting Japanese exchange. We can't read it though, if it were perforated instead of printed in ink, it would apparently make good music on the player-piano.

